# Core Proteomic Analysis of Unique Metabolic Pathways of *Salmonella enterica* for the Identification of Potential Drug Targets

**DOI:** 10.1371/journal.pone.0146796

**Published:** 2016-01-22

**Authors:** Reaz Uddin, Muhammad Sufian

**Affiliations:** 1 Dr. Panjwani Center for Molecular Medicine and Drug Research, International Center for Chemical and Biological Sciences, University of Karachi, Karachi 75270, Pakistan; 2 Prince of Wales Clinical School, Faculty of Medicine, UNSW Australia, Sydney, Australia; Indian Institute of Science, INDIA

## Abstract

**Background:**

Infections caused by *Salmonella enterica*, a Gram-negative facultative anaerobic bacteria belonging to the family of Enterobacteriaceae, are major threats to the health of humans and animals. The recent availability of complete genome data of pathogenic strains of the *S*. *enterica* gives new avenues for the identification of drug targets and drug candidates. We have used the genomic and metabolic pathway data to identify pathways and proteins essential to the pathogen and absent from the host.

**Methods:**

We took the whole proteome sequence data of 42 strains of *S*. *enterica* and *Homo sapiens* along with KEGG-annotated metabolic pathway data, clustered proteins sequences using CD-HIT, identified essential genes using DEG database and discarded *S*. *enterica* homologs of human proteins in unique metabolic pathways (UMPs) and characterized hypothetical proteins with SVM-prot and InterProScan. Through this core proteomic analysis we have identified enzymes essential to the pathogen.

**Results:**

The identification of 73 enzymes common in 42 strains of *S*. *enterica* is the real strength of the current study. We proposed all 73 unexplored enzymes as potential drug targets against the infections caused by the *S*. *enterica*. The study is comprehensive around *S*. *enterica* and simultaneously considered every possible pathogenic strain of *S*. *enterica*. This comprehensiveness turned the current study significant since, to the best of our knowledge it is the first subtractive core proteomic analysis of the unique metabolic pathways applied to any pathogen for the identification of drug targets. We applied extensive computational methods to shortlist few potential drug targets considering the druggability criteria e.g. Non-homologous to the human host, essential to the pathogen and playing significant role in essential metabolic pathways of the pathogen (i.e. *S*. *enterica*). In the current study, the subtractive proteomics through a novel approach was applied i.e. by considering only proteins of the unique metabolic pathways of the pathogens and mining the proteomic data of all completely sequenced strains of the pathogen, thus improving the quality and application of the results. We believe that the sharing of the knowledge from this study would eventually lead to bring about novel and unique therapeutic regimens against the infections caused by the *S*. *enterica*.

## Introduction

*Salmonella enterica* is a Gram-negative facultative anaerobic intracellular bacterium. According to the classification scheme of Kauffmann-White [1], more than 2500 serological variants (or serovars) were categorized in six subspecies [2, 3]. Most of the serovars have a broad range of hosts while some have adapted to specific hosts. The mechanism of adaptation is currently unclear [4]. Typically, *S*. *enterica* serovars infect the host through the mouth, leading to the three major symptoms: enterocolitis, bacteremia and enteric fever, or asymptomatic chronic carriage [5]. Human pathogens include serovar Typhi, Paratyphi, Typhimurium, Sendai, Choleraesuis, Dublin and many others [3].

Pathogenesis of *Salmonella enterica* initiates with its entry in the host organism. Salmonella is usually acquired from the environment by contact with a carrier host or by oral intake of contaminated food or water. After ingestion, Salmonella survives the low pH of the stomach, eventually leading to entry of the intestine where it uses a type III secretion system to deliver effecter proteins essential for intestinal invasion [6]. Hereafter, bacterial progression within the host is different in Non-Typhoidal Salmonella and Typhoidal Salmonella. Non-typhoidal Salmonella serovars induce a localized inflammation which, in immunocompetent persons, results in enterocolitis with the infiltration of polymorphonuclear leukocytes (PMNs) into the sub-mucosal epithelium [7]. In Typhoidal Salmonella, intestinal inflammation is moderate, largely consisting of macrophage infiltration [8] and the bacteria is distributed and reaches the blood either directly or via the mesenteric lymph nodes or are transported within leukocytes, causing bacteremia [9]. Both types of Salmonella grow and persist in systemic tissues where they adapt to the intracellular environment. The pathogen can escape from host cells using secretion systems [10].

A genome is the set of genes in a single functional organism, whereas the pangenome of a prokaryote is the set of non-redundant genes which includes a core genome containing genes present in all strains; dispensable genes that are absent from one or more strains, but not all; and genes that are unique to each strain [11]. Recently, microbial pangenomics has attracted the scientific community which was inspired by the accessibility to sequenced data of whole-genomes of the strains of particular species [12–15]. Simultaneously, research on pan-proteomics was also initiated to study the effects of similarities and differences at the protein level among the strains of specie [16–18]. As of October 13, 2015, there were only 45 target genes reported in DrugBank Database for S. enterica, which covers only 1.6% of its core genome size i.e. 2,800 [19]. Since the pathogen has developed resistance against conventional drugs, so there is a dire need to find new therapeutic drug targets.

In the present study, we took the whole proteome sequence data of 42 strains of 19 serovars of *S*. *enterica* and KEGG-annotated metabolic pathway data of *Homo sapiens*, identified and discarded *S*. *enterica* homologs of human proteins in unique metabolic pathways (UMPs) and identified enzymes essential to the pathogen using DEG database. We compared our results to a previous study [20] where they searched for new antimicrobial targets by focusing on different metabolic enzymes of a single serovar and comparing the results with other serovars at the genome level. In a more recent report, the pangenomic analyses of 22 complete and 23 draft genome sequences was performed [19]. However, to the best of our knowledge the current study is the first subtractive core proteomic analysis of the unique metabolic pathways applied to any pathogen for the identification of drug targets primarily essential enzymes.

## Methodology

**Fig 1:**
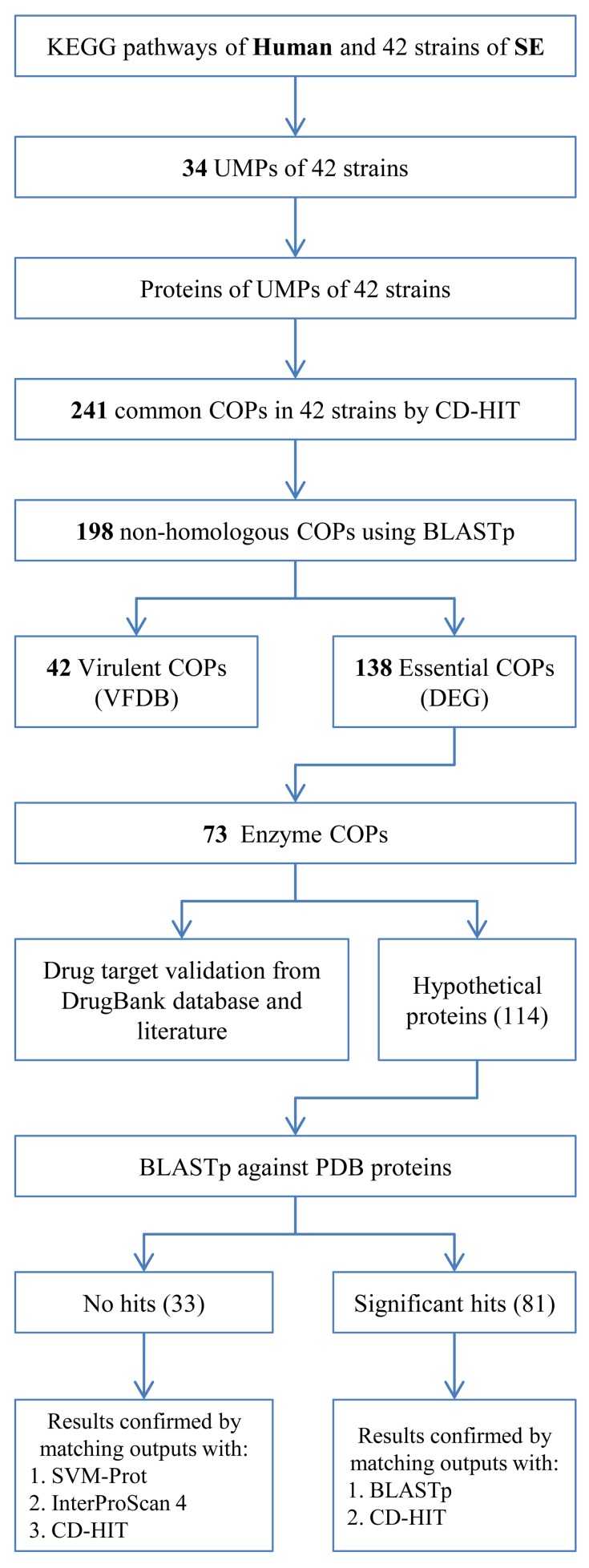
Methodology

A schematic representation of the methodology is given in Fig 1. 88 biological datasets used in our analyses were downloaded from online sources, details of which are given in S1 Table.

### 1. Identification of UMPs of *S*. *enterica*

KEGG Brite Hierarchy files of *H*. *sapiens* and 42 strains of *S*. *enterica* containing information about the genes of respective metabolic pathways were downloaded from the KEGG database [21]. The metabolic pathways unique to the serovars (i.e. missing in human host) were identified using KEGG Orthology (KO) IDs, and the corresponding genes were sorted out. The UMPs absent in some strains were listed out using in-house AWK scripts.

### 2. Clustering common proteins of UMPs of 42 strains

The KEGG IDs of all the genes from UMPs were converted to corresponding NCBI GIs using KEGG-API service [21]. Amino acid sequences were retrieved from the respective strains available on NCBI FTP server [22] using Fastblast [23]. The genes encoding tRNA and rRNA were excluded since the aim was to propose enzymes as the drug targets. Further plasmid-encoded genes were not considered to be essential for the survival of cell, as per information available in the Database of Essential Genes (DEG) [24]. We noticed that some NCBI GIs were discontinued and therefore, updated to the new GIs. We linked the new GIs with the old one and retrieved the sequence. CD-HIT [25] is a standalone command-based application which groups a set of sequences of a database on the basis of sequence identity. Orthologs within the 42 strains were identified by using CD-HIT (updated on August 27, 2012) to group protein sequences with at least 80% sequence identity in to Clusters of Proteins (COPs) so that each COP will be analyzed at once for further steps of subtractive proteomics. The results were verified by comparison to the online server of ElimDupes [26].

### 3. Searching of non-homologous essential enzymes

To process all COPs for subtractive proteomic analyses at once, a novel strategy was applied which comprised of two approaches. In first approach, proteins of all COPs were subjected to BLASTp [27] against *Homo sapiens* downloaded from NCBI FTP server [28] and the output was analyzed for non-homologous proteins. In second approach, 3 strains out of 42 were selected at random and proteins of those strains were subjected to BLASTp against human proteome. Both approaches are illustrated in S1 Fig. The parameter details for BLASTp are mentioned in Table 1 (a). The results of both approaches were observed by BioPerl module SearchIO [29] and the better approach was adapted to the next steps considering the criteria of time processing. The non-homologous COPs from the previous step were subjected to BLASTp of DEG V. 10 [24] to identify essential genes of the pathogen. The parameter details are mentioned in Table 1 (b). The KEGG Brite hierarchy is one of the important features of KEGG server containing the information of enzymes of metabolic pathways. The enzymes were sorted out from non-homologous essential COPs of *S*. *enterica* using the hierarchy files of 42 strains [21].

**Table 1:** Parameters for BLASTp.

	a	b	c	d
**Program**	BLAST+ 2.2.28	BLASTp of DEG 10	BLAST+ 2.2.28	BLAST+ 2.2.28
**Query name**	COPs	Non-homologous COPs	Non-homologous COPs	Hypothetical proteins
**No. of queries**	241	198	198	114
**Subject name**	Human proteome	DEG	VFDB	PDB proteins
**No. of subjects**	68,939	12,379	2,447	252,484
**E-value**	1.00E-03	1.00E-05	1.00E-04	1.00E-05

### 4. Searching the virulent genes

VFDB (Virulence Factors Database) [30]containing protein sequences of all virulent genes was downloaded and non-homologous COPs from 3 randomly selected strains were subjected to standalone BLASTp against VFDB sequences to find out virulent genes with sequence identity of 70% or more. Table 1 (c) contained the parameter details.

### 5. Characterization of the hypothetical proteins

The hypothetical proteins were identified among the enzymes to characterize their structure and/or function. All the hypothetical protein sequences were subjected to standalone BLASTp against protein sequences available in PDB (Protein Data Bank) [31] obtained from PDB FTP server [32]. The parameter details are mentioned in Table 1 (d). The queries with significant hits against PDB database were verified from CD-HIT output and those with ‘no hits’ were subjected to SVM-Prot [33] and InterProScan version 4.0 [34] for protein family prediction. The results were manually cross-checked with CD-HIT output.

### 6. Validation from the literature:

The non-homologous catalytic proteins considered as putative drug targets were validated from DrugBank database [35] and published results of Becker et. al. [20]. In order to do so, the gene symbols of essential enzymes [20] were converted to full form using DAVID Bioinformatics tool [36], and then searched in both sources manually.

## Results and Discussion

### 1. Identification of UMPs of *S*. *enterica*

**Fig 2:**
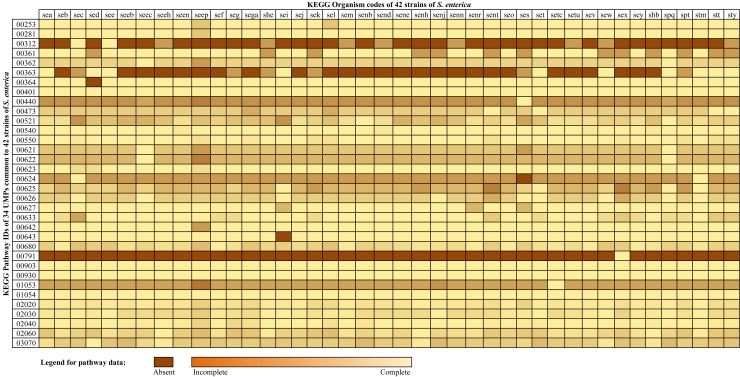
Heatmap of genes in UMPs of *S*. *enterica* strains. The heatmap contains percentage presence and absence of genes of in each metabolic pathway of 42 strains of *S*. *enterica*.

Each of the metabolic pathways of 42 strains of the *S*. *enterica* was compared with the complete human metabolic pathway. On average, each strain has 117 metabolic pathways and at least 34 UMPs (Table 2) with all UMPs present in almost all strains. A heatmap containing the percentage presence of proteins in each pathway and totally absent pathways in individual strains is illustrated in Fig 2, while its corresponding quantitative data is provided as S2 Table. In the studied strains of *S*. *enterica*, we found that only the strain (Typhi P-stx-12) was predicted to metabolize the Atrazine, thus may be resistant to it. However the dataset lacked the pathway information of β-Lactam resistance and Bisphenol degradation which were also the next most frequent absent pathways among all studied strains. The strains Heidelberg CFSAN002069 and Typhi CT18 needed to update in KEGG since the data was not updated and hence 22 and 11 NCBI GIs were appended, respectively in both strains and mentioned in S3 Table.

**Table 2:** Details of Metabolic Pathways and Genes of human and 42 strains of *S*. *enterica*.

S.No.	Organism name	Organism KEGG Code	No. of Pathways	Unique Pathways	KEGG ID	NCBI RefSeq ID	NCBI Gis	Sequences
	Homo sapiens	has	286	-	-	H_sapiens	-	-
1	Agona SL483	sea	118	32	428	NC_011149	426	417
2	Arizonae 62 z4 z23	ses	117	31	407	NC_010067	406	406
3	Bareilly CFSAN000189	see	119	33	429	NC_021844	428	426
4	Bovismorbificans 3114	senb	117	31	414	NC_022241	414	414
5	Choleraesuis SC B67	sec	119	33	410	NC_006905	410	408
6	Cubana CFSAN002050	seeb	117	31	416	NC_021818	416	415
7	Dublin CT 02021853	sed	116	31	425	NC_011205	425	416
8	Enteritidis P125109	setc	117	31	435	NC_011294	435	430
9	Gallinarum 287 91	sega	116	31	399	NC_011274	399	399
10	Gallinarum Pullorum CDC1983 67	seg	117	32	409	NC_022221	409	409
11	Gallinarum pullorum RKS5078	sel	116	31	395	NC_016831	395	395
12	Heidelberg 41578	seec	117	31	430	NC_021810	430	426
13	Heidelberg B182	shb	116	31	442	NC_017623	440	430
14	Heidelberg CFSAN002069	senh	116	31	451	NC_021812	451	440
15	Heidelberg SL476	seh	119	33	433	NC_011083	431	431
16	Javiana CFSAN001992	senj	115	31	419	NC_020307	419	419
17	Newport SL254	seeh	118	32	444	NC_011080	444	433
18	Newport USMARC S3124 1	senn	118	32	425	NC_021902	425	425
19	Paratyphi A AKU 12601	sek	117	32	405	NC_011147	404	404
20	Paratyphi A ATCC 9150	spt	117	32	408	NC_006511	407	407
21	Paratyphi B SPB7	spq	118	32	428	NC_010102	427	427
22	Paratyphi C RKS4594	sei	116	31	418	NC_012125	418	418
23	Pullorum S06004	seep	116	31	375	NC_021984	375	375
24	Schwarzengrund CVM19633	sew	119	33	436	NC_011094	435	424
25	Thompson RM6836	sene	117	31	421	NC_022525	421	421
26	Typhi CT18	sty	119	33	409	NC_003198	409	408
27	Typhi P stx 12	sex	117	32	407	NC_016832	407	406
28	Typhi Ty2	stt	117	32	409	NC_004631	409	409
29	Typhi Ty21a	sent	116	31	406	NC_021176	406	406
30	Typhimurium 08–1736	seen	117	31	420	NC_021820	420	420
31	Typhimurium 14028S	seo	117	31	433	NC_016856	433	433
32	Typhimurium 798	sef	117	31	430	NC_017046	430	430
33	Typhimurium D23580	sev	117	31	434	NC_016854	434	434
34	Typhimurium DT104	send	119	32	426	NC_022569	426	426
35	Typhimurium DT2	senr	117	31	428	NC_022544	428	428
36	Typhimurium LT2	stm	118	32	447	NC_003197	447	447
37	Typhimurium SL1344	sey	117	31	435	NC_016810	435	435
38	Typhimurium ST4 74	seb	117	31	437	NC_016857	437	437
39	Typhimurium T000240	sem	119	32	438	NC_016860	438	438
40	Typhimurium U288	setu	119	32	434	NC_021151	434	433
41	Typhimurium UK 1	sej	117	31	430	NC_016863	430	430
42	Typhimurium var 5 CFSAN001921	set	117	32	422	NC_021814	422	422

### 2. Clustering common proteins of UMPs of 42 strains and searching of non-homologous essential enzymes

The CD-HIT resulted in 537 COPs and each cluster was comprised of more than 1 protein. Out of total, 241 COPs contained at least 42 proteins belonging to the 42 strains of *S*. *enterica*. S4 Table contained the NCBI-GIs of orthologous proteins (genes) clustered in groups.

The complete human proteome was obtained from NCBI FTP server (details in S1 Table). The non-homologous proteins could be potential drug targets with reduced possible side effects or cross reactivity of the drug with the host proteins. It is essential to find the similarity of the shortlisted sequences with the human host. In order to do so, we compared each COP with the individual human proteins. We performed this comparison by two separate approaches (details in methods section). As stated earlier that the COPs were consisted of up to 80% similar proteins; therefore, if we compare either (i) each single entry of the COPs with the host proteins or (ii) comparing few randomly selected entries of the COPs with human host proteins, the outcome would remain same. We used both of the approaches to see if the statement maintains. Both approaches of searching non-homologous sequences in the pathogen revealed exactly same results i.e. 198 out of 241 COPs were identified as non-homologous to humans (Table 3). The second approach was selected for the further steps of subtractive proteomics as the approach was accurate and relatively fast. The COP names mentioned in Table 3 were allocated by the authors following the criteria of maximum or common occurrences of that name in a respective cluster. One important aspect was observed during the tabulation of data (Table 3) that despite having exactly the same or closely similar names within the COPs, the member proteins of the respective COPS showed low similarity among them. These COPs include Cytochrome BD-II Ubiquinol Oxidase (COP # 139 and 221), D-alanyl-D-alanine Carboxypeptidase (COP # 127 and 190), Lipopolysaccharide core biosynthesis protein (COP # 250, 339 and 384), Peptidoglycan Synthetase FtsI (COP # 65 and 67), PTS system Ascorbate-specific transporter IIC (COP # 129 and 164), Transcriptional regulator (COP # 17 and 167), Tricarboxylate transport membrane protein (COP # 109 and 476), Two component response regulator (COP # 378, 410 and 411) and Type III Secretion apparatus protein SpaR (COP # 341 and 344). From the similar named COPs, we randomly selected the few proteins and subjected to online BLASTp which resulted in low similarity in each case. There might be two possibilities for the outcome; either these sets of COPs were isozymes or might be human error during the GenBank submission. For instance BLASTp of NCBI GI 194443076 and 194443845 have only 29% identity though they both have same name and belong to the same strain. The beta subunit of the subtype 1 and 2 of the enzyme *Nitrate reductase* shared more than 80% sequence similarity and hence clustered in a single COP. The enzyme *Succinate Dehydrogenase* Cytochrome b556 large membrane was somehow not characterized as an enzyme during KEGG analysis hence its UniProt ID was mentioned in Table 3.

**Table 3:** Functional characterization of non-homologous COPs.

COP Name	Subtype	COP #	Virulent	Essential	Enzyme	Becker 2006
[Citrate (pro-3S)-lyase] ligase		247				
2-(5''-triphosphoribosyl)-3'-dephospho-CoA synthase		432				
2-dehydro-3-deoxyphosphooctonate aldolase		328		Yes	Yes	Yes
3-deoxy-D-manno-octulosonic-acid transferase		192		Yes	Yes	Yes
3-deoxy-manno-octulosonate cytidylyltransferase		361		Yes	Yes	Yes
Acetate kinase		205		Yes	Yes	
ADP-heptose—LPS heptosyltransferase	I	291		Yes	Yes	Yes
	II	261		Yes	Yes	Yes
Aerotaxis receptor		104		Yes		
Alanine racemase		245		Yes	Yes	
Alkylphosphonate utilization operon protein PhnA		498		Yes	Yes	
Anti-sigma-28 factor	FlgM	507				
Aspartate racemase		366				
Bifunctional chorismate mutase/prephenate dehydrogenase		227				
Carbon storage regulator		527		Yes		
Chemotaxis methyltransferase	CheR	321		Yes	Yes	
Chemotaxis protein	CheA	49		Yes	Yes	Dispensable
	CheW	276		Yes		
	CheZ	409				
	CheY	486	Yes	Yes		
Chemotaxis-specific methylesterase		259	Yes	Yes	Yes	
Chromosomal replication initiation protein		136		Yes		
Citrate lyase	Gamma	505		Yes	Yes	
Colanic acid capsular biosynthesis activation protein	A	416				
Cytochrome BD-II ubiquinol oxidase	1	221		Yes	Yes	
	1	139		Yes	Yes	
	2	273		Yes	Yes	
D-alanyl-D-alanine carboxypeptidase		127		Yes	Yes	
		190		Yes	Yes	
DNA-binding transcriptional activator	DcuR	376		Yes		
	KdpE	395		Yes		
	SdiA	355				
	UhpA	404		Yes		
DNA-binding transcriptional regulator	BaeR	374		Yes		
	BasR	390	Yes	Yes		
	CpxR	385		Yes		
	PhoP	398	Yes	Yes		
	QseB	336		Yes		
	RstA	368		Yes		
D-ribose transporter	RbsB	310		Yes		
Flagella synthesis protein	FlgN	478				
Flagellar assembly protein	FliH	370				
Flagellar basal body L-ring protein		382	Yes			
Flagellar basal body P-ring biosynthesis protein	FlgA	401				
Flagellar basal body rod modification protein		383				
Flagellar basal body rod protein	FlgB	479	Yes			
	FlgC	484	Yes			
	FlgF	354				
	FlgG	343	Yes			
Flagellar biosynthesis protein	FliJ	471		Yes		
	FliO	487		Yes		
	FliP	364	Yes			
	FliQ	517	Yes			
	FliR	340				
	FliT	490				
Flagellar hook protein	FlgE	201				
Flagellar hook-associated protein	FlgL	290		Yes		
Flagellar hook-basal body protein	FliE	503				
Flagellar hook-length control protein		199				
Flagellar motor protein	MotA	312		Yes		
Flagellar motor switch protein	FliM	275	Yes			
Flagellar motor switch protein	G	279	Yes			
Flagellar MS-ring protein		68				
Flagellar protein	FliS	481	Yes			
Formate dehydrogenase-O	Gamma	412				
Fructose 1,6-bisphosphate aldolase		244		Yes	Yes	
Fumarate reductase	C	485				
	D	492		Yes		
Glutamate/aspartate ABC transporter permease	GltK	397		Yes		
Hydrogenase 2	Large	72				
	Small	230		Yes	Yes	
Integral membrane protein	MviN	90		Yes		
Invasion protein	InvA	48	Yes			
Isochorismatase		326	Yes	Yes	Yes	
Isochorismate synthase		174		Yes	Yes	
Lipid A biosynthesis lauroyl acyltransferase		280		Yes	Yes	Yes
Lipid-A-disaccharide synthase		218		Yes	Yes	Yes
Lipopolysaccharide 1,2-glucosyltransferase		272		Yes	Yes	
Lipopolysaccharide 1,3-galactosyltransferase		271		Yes	Yes	
Lipopolysaccharide core biosynthesis protein		250		Yes	Yes	
		339		Yes	Yes	Yes
		384		Yes	Yes	
	RfaG	226		Yes	Yes	
Maltose ABC transporter substrate-binding protein		132		Yes		
Monofunctional biosynthetic peptidoglycan transglycosylase		369		Yes	Yes	
Multidrug efflux system	MdtC	12		Yes		
Nitrate reductase 1	Alpha	4		Yes	Yes	
Nitrate reductase molybdenum cofactor assembly chaperone 1		381				
Nitrate reductase (81 duplicates of 1 and 2)	Beta	98		Yes	Yes	
Nitrogen regulation protein	NR(I)	135		Yes		
	NR(II)	260		Yes	Yes	
	P-II 1	497		Yes		
O-antigen ligase		166		Yes	Yes	Yes
Osmolarity response regulator	OmpR	367		Yes		
Osmolarity sensor protein	EnvZ	160		Yes	Yes	
Outer membrane channel protein	TolC	120		Yes		
Outer membrane lipoprotein		482				
Outer membrane porin protein	C	223		Yes		
Outer membrane protease		293	Yes			
Outer membrane protein	F	238		Yes		
Penicillin-binding protein	1b	33		Yes	Yes	Yes
	2	54		Yes		
Peptide transport periplasmic protein	SapA	76		Yes		
Peptidoglycan synthetase	1a	31		Yes	Yes	Yes
	FtsI	65		Yes	Yes	Yes
	FtsI	67		Yes	Yes	
Phosphate ABC transporter substrate-binding protein		251		Yes		
Phosphate acetyltransferase		43		Yes	Yes	
Phosphate regulon sensor protein	PhoR	186		Yes	Yes	
Phosphoenolpyruvate carboxylase		29		Yes	Yes	
Phosphoenolpyruvate-protein phosphotransferase		40		Yes	Yes	
Phosphoglyceromutase		93		Yes	Yes	
Phospho-N-acetylmuramoyl-pentapeptide-transferase		242		Yes	Yes	Yes
PII uridylyl-transferase		23		Yes	Yes	
Preprotein translocase	SecA	22		Yes		
	SecB	459		Yes		
	SecD	60		Yes		
	SecE	477		Yes		
	SecF	270		Yes		
	SecG	439		Yes		
	SecY	169		Yes		
	YajC	500		Yes		
PTS system ascorbate-specific transporter	IIC	129		Yes		
	IIC	164		Yes		
PTS system fructose-specific transporter	IIBC	74		Yes	Yes	
PTS system glucitol/sorbitol-specific transporter	IIA	491				
	IiB	284				
PTS system glucose-specific transporter	IIA	447		Yes	Yes	
	IIBC	119		Yes	Yes	
PTS system lactose/cellobiose-specific transporter	IIB	515				
PTS system L-ascorbate-specific transporter	IIA	460		Yes	Yes	
PTS system mannitol-specific transporter	IIA	465		Yes	Yes	
	IIABC	50		Yes	Yes	
PTS system mannose-specific transporter	IiAB	285		Yes	Yes	
	IIC	338				
	IID	324				
PTS system N,N'-diacetylchitobiose-specific transporter	IIA	496		Yes	Yes	
	IIB	499		Yes	Yes	
	IIC	158				
PTS system phosphohistidinoprotein-hexose phosphotransferase	Hpr	514		Yes		
	Npr	516		Yes		
PTS system transporter subunit IIA-like nitrogen-regulatory protein	PtsN	452		Yes	Yes	
Purine-binding chemotaxis protein		449	Yes	Yes		
Respiratory nitrate reductase 1	Gamma	396				
RNA polymerase sigma factor for flagellar biosynthesis		377	Yes	Yes		
RNA polymerase sigma-54 factor		128		Yes		
Sec-independent translocase		434				
Secretion system apparatus protein	SsaU	255	Yes			
	SsaV	45	Yes			
Sensor protein	PhoQ	123	Yes	Yes	Yes	
	BasS/ PmrB	246	Yes	Yes	Yes	
	RstB	179		Yes	Yes	
Signal transduction histidine-protein kinase	BaeS	140		Yes	Yes	
Succinate dehydrogenase cytochrome b556 large membrane		483		Yes	K8TKP2	
Surface presentation of antigens protein	SpaO	305	Yes			
	SpaP	400	Yes			
	SpaQ	521	Yes	Yes		
	SpaS	249	Yes			
Tetraacyldisaccharide 4'-kinase		282		Yes	Yes	Yes
Tetrathionate reductase complex	A	13	Yes			
Transcriptional activator	FlhC	423	Yes			
	FlhD	494	Yes			
Transcriptional regulator	PhoB	387		Yes		
		167		Yes		
		17	Yes	Yes		
	RcsB	386		Yes		
Tricarboxylate transport membrane protein		109				
		476				
Twin arginine translocase	A	522		Yes		
	E	525		Yes		
Twin-arginine protein translocation system	TatC	345		Yes		
Two component response regulator		410	Yes	Yes		
		411	Yes	Yes		
		378		Yes		
Two-component sensor kinase protein		152		Yes	Yes	
Type III secretion apparatus lipoprotein YscJ/HrcJ family		352	Yes	Yes		
Type III secretion apparatus needle protein	PrgI	523	Yes			
	SsaG	519	Yes			
Type III secretion apparatus protein	SpaR	341	Yes	Yes		
	SpaR	344	Yes	Yes		
Type III secretion outer membrane pore		111	Yes			
Type III secretion outer membrane protein YscC/HrcC family		73	Yes			
Type III secretion system protein		286	Yes	Yes		
	FliP	407	Yes			
	InvE	229	Yes			
UDP pyrophosphate phosphatase		333		Yes	Yes	
UDP-2,3-diacylglucosamine hydrolase		372		Yes	Yes	Yes
UDP-3-O-[3-hydroxymyristoyl] glucosamine N-acyltransferase		265		Yes	Yes	Yes
UDP-3-O-[3-hydroxymyristoyl] N-acetylglucosamine deacetylase		301		Yes	Yes	Yes
UDP-N-acetylenolpyruvoylglucosamine reductase		263		Yes	Yes	Yes
UDP-N-acetylglucosamine 1-carboxyvinyltransferase		194		Yes	Yes	Yes
UDP-N-acetylglucosamine acyltransferase		342		Yes	Yes	Yes
UDP-N-acetylmuramate—L-alanine ligase		121		Yes	Yes	Yes
UDP-N-acetylmuramoylalanyl-D-glutamate—2,6-diaminopimelate ligase		113		Yes	Yes	Yes
UDP-N-acetylmuramoyl-L-alanyl-D-glutamate synthetase		177		Yes	Yes	Yes
UDP-N-acetylmuramoyl-tripeptide—D-alanyl-D-alanine ligase		157		Yes	Yes	Yes
Virulence membrane protein	PagC	430				
Zinc resistance protein		467		Yes		

**Fig 3:**
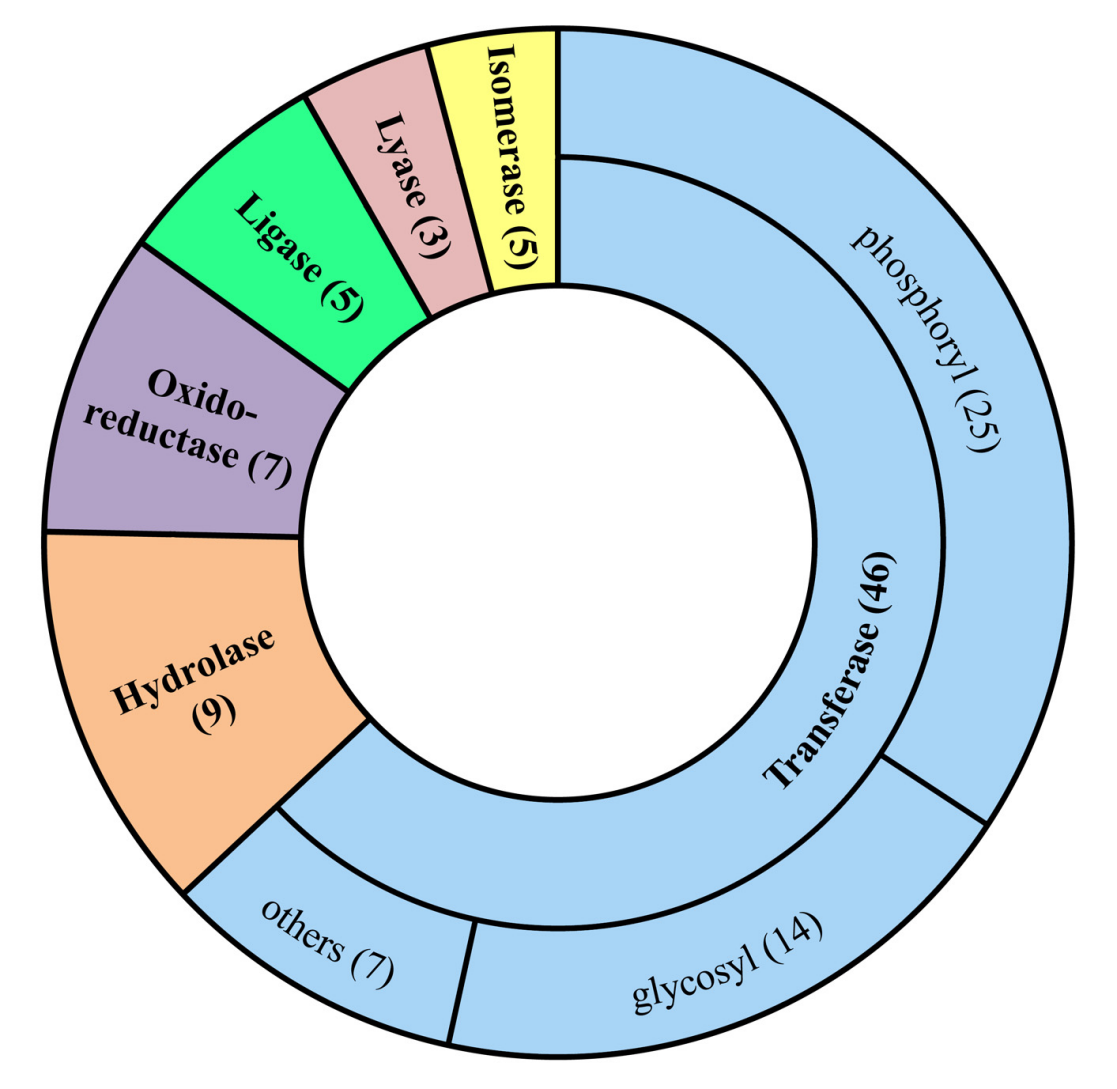
Enzyme classification of 73 potential drug targets. The pie chart reveals that 63% of the enzyme targets belong to Transferase class which is subdivided into phosphoryl (34%), glycosyl (19%) and other (10%) transferases.

Additionally, we searched for the essential and virulent genes from the 198 COPs by applying the same subtractive proteomics approach. The database of essential genes (DEG) is a well curated open-access database consisting of essential genes from various organisms ranging from single-cell prokaryotes to multicellular eukaryotes. The bacteria harbor various virulent genes which lead to pathogenecity. Therefore, identifying virulent factors in the genome could lead us to elucidate the molecular mechanism of bacterial pathogenecity. The VFDB [30] is an online server containing information about virulent genes present in various microorganisms. Similar results were obtained from 3 randomly selected strains and it was found out that 138 out of 198 COPs were essential for the bacteria as per the prediction of DEG (Table 3), and 42 out of 198 COPs were identified as virulent genes (Table 3). There were 73 enzymes in the 138 non-humongous essential COPs (Table 3). The NCBI GIs of each respective COP was presented in S5 Table. The S1 Text contained important information regarding the accessibility of NCBI GIs mentioned in S5 Table. The data illustrated through pie chart in Fig 3 and tabulated in Table 4 revealed that most of the targets (34%) belonged to the subclass ‘phosphoryl transferases’ or ‘kinases’ which are the most favorable targets in drug discovery research [37].

**Table 4:** Enzyme Classification of 73 drug targets.

Enzyme name	E.C. Number	Enzyme Class	Enzyme Sub-class
Cytochrome BD-II ubiquinol oxidase 1	1.10.3.10	Oxidoreductase	diphenols as donors
Cytochrome BD-II ubiquinol oxidase 2	1.10.3.10	Oxidoreductase	diphenols as donors
Cytochrome BD-II ubiquinol oxidase 3	1.10.3.10	Oxidoreductase	diphenols as donors
Hydrogenase 3	1.12.-.-	Oxidoreductase	hydrogen as donor
UDP-N-acetylenolpyruvoylglucosamine reductase	1.3.1.98	Oxidoreductase	CH-CH group of donors
Nitrate reductase 1	1.7.99.4	Oxidoreductase	nitrogenous compounds as donors
Nitrate reductase 2	1.7.99.4	Oxidoreductase	nitrogenous compounds as donors
Chemotaxis methyltransferase	2.1.1.80	Transferase	One-Carbon group
Lipid A biosynthesis lauroyl acyltransferase	2.3.1.-	Transferase	acyl
UDP-N-acetylglucosamine acyltransferase	2.3.1.129	Transferase	acyl
UDP-3-O-[3-hydroxymyristoyl] glucosamine N-acyltransferase	2.3.1.191	Transferase	acyl
Phosphate acetyltransferase	2.3.1.8	Transferase	acyl
ADP-heptose—LPS heptosyltransferase 1	2.4.-.-	Transferase	glycosyl
ADP-heptose—LPS heptosyltransferase 2	2.4.-.-	Transferase	glycosyl
Lipopolysaccharide core biosynthesis protein 1	2.4.-.-	Transferase	glycosyl
Lipopolysaccharide core biosynthesis protein 2	2.4.-.-	Transferase	glycosyl
Lipopolysaccharide core biosynthesis protein 3	2.4.-.-	Transferase	glycosyl
Lipopolysaccharide core biosynthesis protein 4	2.4.-.-	Transferase	glycosyl
Peptidoglycan synthetase 1	2.4.1.129	Transferase	glycosyl
Peptidoglycan synthetase 2	2.4.1.129	Transferase	glycosyl
Peptidoglycan synthetase 3	2.4.1.129	Transferase	glycosyl
Lipid-A-disaccharide synthase	2.4.1.182	Transferase	glycosyl
Lipopolysaccharide 1,3-galactosyltransferase	2.4.1.44	Transferase	glycosyl
Lipopolysaccharide 1,2-glucosyltransferase	2.4.1.58	Transferase	glycosyl
Monofunctional biosynthetic peptidoglycan transglycosylase	2.4.2.-	Transferase	glycosyl
3-deoxy-D-manno-octulosonic-acid transferase	2.4.99.12	Transferase	glycosyl
2-dehydro-3-deoxyphosphooctonate aldolase	2.5.1.55	Transferase	alkyl
UDP-N-acetylglucosamine 1-carboxyvinyltransferase	2.5.1.7	Transferase	alkyl
Tetraacyldisaccharide 4'-kinase	2.7.1.130	Transferase	phosphorus
PTS system fructose-specific transporter	2.7.1.69	Transferase	phosphorus
PTS system glucose-specific transporter 1	2.7.1.69	Transferase	phosphorus
PTS system glucose-specific transporter 2	2.7.1.69	Transferase	phosphorus
PTS system L-ascorbate-specific transporter	2.7.1.69	Transferase	phosphorus
PTS system mannitol-specific transporter 1	2.7.1.69	Transferase	phosphorus
PTS system mannitol-specific transporter 2	2.7.1.69	Transferase	phosphorus
PTS system mannose-specific transporter	2.7.1.69	Transferase	phosphorus
PTS system N,N'-diacetylchitobiose-specific transporter 1	2.7.1.69	Transferase	phosphorus
PTS system N,N'-diacetylchitobiose-specific transporter 2	2.7.1.69	Transferase	phosphorus
PTS system transporter subunit IIA-like nitrogen-regulatory protein	2.7.1.69	Transferase	phosphorus
Chemotaxis protein	2.7.13.3	Transferase	phosphorus
Osmolarity sensor protein	2.7.13.3	Transferase	phosphorus
Phosphate regulon sensor protein	2.7.13.3	Transferase	phosphorus
Sensor protein 1	2.7.13.3	Transferase	phosphorus
Sensor protein 2	2.7.13.3	Transferase	phosphorus
Sensor protein 3	2.7.13.3	Transferase	phosphorus
Signal transduction histidine-protein kinase	2.7.13.3	Transferase	phosphorus
Acetate kinase	2.7.2.1	Transferase	phosphorus
Nitrogen regulation protein	2.7.3.-	Transferase	phosphorus
Two-component sensor kinase protein	2.7.3.-	Transferase	phosphorus
Phosphoenolpyruvate-protein phosphotransferase	2.7.3.9	Transferase	phosphorus
3-deoxy-manno-octulosonate cytidylyltransferase	2.7.7.38	Transferase	phosphorus
PII uridylyl-transferase	2.7.7.59	Transferase	phosphorus
Phospho-N-acetylmuramoyl-pentapeptide-transferase	2.7.8.13	Transferase	phosphorus
Chemotaxis-specific methylesterase	3.1.1.61	Hydrolase	Ester bond
Alkylphosphonate utilization operon protein PhnA	3.11.1.2	Hydrolase	phosphonoacetate
Isochorismatase	3.3.2.1	Hydrolase	Ether bond
D-alanyl-D-alanine carboxypeptidase 1	3.4.16.4	Hydrolase	peptidase
D-alanyl-D-alanine carboxypeptidase 2	3.4.16.4	Hydrolase	peptidase
Penicillin-binding protein	3.4.16.4	Hydrolase	peptidase
UDP-3-O-[3-hydroxymyristoyl] N-acetylglucosamine deacetylase	3.5.1.-	Hydrolase	linear amides
UDP pyrophosphate phosphatase	3.6.1.27	Hydrolase	acid anhydrides
UDP-2,3-diacylglucosamine hydrolase	3.6.1.54	Hydrolase	acid anhydrides
Phosphoenolpyruvate carboxylase	4.1.1.31	Lyase	Carbon-Carbon
Fructose 1,6-bisphosphate aldolase	4.1.2.13	Lyase	Carbon-Carbon
Citrate lyase	4.1.3.6	Lyase	Carbon-Carbon
Alanine racemase	5.1.1.1	Isomerase	Epimerases
Phosphoglyceromutase	5.4.2.-	Isomerase	Intramolecular transfer
Isochorismate synthase	5.4.99.6	Isomerase	Intramolecular transfer
O-antigen ligase	6.-.-.-	Ligase	Ligase
UDP-N-acetylmuramoyl-tripeptide—D-alanyl-D-alanine ligase	6.3.2.10	Ligase	Peptide Synthases
UDP-N-acetylmuramoylalanyl-D-glutamate—2,6-diaminopimelate ligase	6.3.2.13	Ligase	Peptide Synthases
UDP-N-acetylmuramate—L-alanine ligase	6.3.2.8	Ligase	Peptide Synthases
UDP-N-acetylmuramoyl-L-alanyl-D-glutamate synthetase	6.3.2.9	Ligase	Peptide Synthases

### 3. Characterization of the hypothetical proteins

Hypothetical proteins are those for which the sequences are available but their family and functional classification has not been established. As such they may represent unidentified drug targets [38, 39]. The computational methods (for e.g. Blast2GO, HMMscan, KEGG Automatic Annotation Server (KAAS), ProtParam server, PSORTb, SVMProt, etc) are effective in annotating the functional and family classes of the big number of hypothetical sequences present in bacterial genomes [40–42]. The functional classification may lead us to predict the mechanism of the possible metabolic pathway in which the protein is involved. In order to characterize the hypothetical proteins among the shortlisted COPs, we first looked how many proteins were hypothetical. We found out that there were 3,105 proteins in 73 COPs, out of which 114 proteins were hypothetical (Table 5). The identifier details of these 3,105 enzymes are provided in S6 Table.

**Table 5:** BLASTp of Hypothetical Proteins in non-homologous COPs in PDB.

NCBI-GI	Protein name	COP #	KEGG Organism code	NCBI RefSeq ID	PDB Best Hit		
					PDB ID	Bit Score	Percent identity
161503125	SARI_01190	4	ses	NC_010067	1q16_A	1247	95.1
161613744	SPAB_01469	4	spq	NC_010102	1q16_A	1247	95.3
538362953	BN855_24210	43	senb	NC_022241	1xco_F	313	46.6
378959111	STBHUCCB_10250	49	sex	NC_016832	2lp4_A	227	84.1
538362544	BN855_20090	49	senb	NC_022241	2lp4_A	227	84.1
161505779	SARI_03955	50	ses	NC_010067	1j6t_A	146	97.9
161616759	SPAB_04578	50	spq	NC_010102	1j6t_A	146	97.9
161504756	SARI_02879	65	ses	NC_010067	4kqr_B	549	45.7
161612466	SPAB_00156	65	spq	NC_010102	4kqr_B	549	45.7
161503040	SARI_01104	67	ses	NC_010067	4kqr_B	539	43.0
161613654	SPAB_01378	67	spq	NC_010102	4kqr_B	539	43.4
538362430	BN855_18930	67	senb	NC_022241	4kqr_B	539	43.4
161503126	SARI_01191	98	ses	NC_010067	3ir7_B	511	92.8
161613745	SPAB_01470	98	spq	NC_010102	3ir7_B	511	93.2
161613976	SPAB_01714	98	spq	NC_010102	3ir7_B	506	80.2
378984138	STMDT12_C15970	98	sem	NC_016860	3ir7_B	506	80.0
538360694	BN855_1290	113	senb	NC_022241	1e8c_B	471	92.4
538361709	BN855_11640	119	senb	NC_022241	1o2f_B	90	93.3
161504450	SARI_02563	139	ses	NC_010067	No hit	-	-
161615466	SPAB_03237	139	spq	NC_010102	No hit	-	-
538362753	BN855_22200	140	senb	NC_022241	4i5s_B	226	30.5
378961722	STBHUCCB_37440	152	sex	NC_016832	4i5s_B	224	31.3
538364097	BN855_35800	160	senb	NC_022241	1bxd_A	161	90.7
29144086	t3806	166	stt	NC_004631	No hit	-	-
62182206	SC3636	166	sec	NC_006905	No hit	-	-
161505752	SARI_03928	166	ses	NC_010067	No hit	-	-
161616791	SPAB_04610	166	spq	NC_010102	No hit	-	-
488656245	TY21A_19335	166	sent	NC_021176	No hit	-	-
378959497	STBHUCCB_14250	179	sex	NC_016832	4i5s_B	222	27.9
538360809	BN855_2450	218	senb	NC_022241	No hit	-	-
161504097	SARI_02195	221	ses	NC_010067	No hit	-	-
161615026	SPAB_02786	221	spq	NC_010102	No hit	-	-
161505743	SARI_03919	226	ses	NC_010067	2iw1_A	374	85.8
161616800	SPAB_04619	226	spq	NC_010102	2iw1_A	374	86.4
538364332	BN855_38170	226	senb	NC_022241	2iw1_A	374	86.1
378962383	STBHUCCB_44400	246	sex	NC_016832	4i5s_B	225	29.8
538364321	BN855_38060	261	senb	NC_022241	1psw_A	346	92.5
161505747	SARI_03923	271	ses	NC_010067	1ss9_A	273	26.0
161616796	SPAB_04615	271	spq	NC_010102	1ga8_A	273	26.0
161505748	SARI_03924	272	ses	NC_010067	3tzt_B	252	27.0
161616795	SPAB_04614	272	spq	NC_010102	3tzt_B	252	25.8
161504449	SARI_02562	273	ses	NC_010067	No hit	-	-
161615465	SPAB_03236	273	spq	NC_010102	No hit	-	-
378960680	STBHUCCB_26520	273	sex	NC_016832	No hit	-	-
379699575	STM474_0375	273	seb	NC_016857	No hit	-	-
538361476	BN855_9270	282	senb	NC_022241	4itn_A	316	27.5
161503046	SARI_01110	285	ses	NC_010067	2jzh_A	170	94.7
161613660	SPAB_01384	285	spq	NC_010102	2jzh_A	170	95.3
378959115	STBHUCCB_10290	321	sex	NC_016832	1af7_A	274	99.3
161504232	SARI_02339	326	ses	NC_010067	2fq1_B	285	87.4
161615198	SPAB_02966	326	spq	NC_010102	2fq1_B	285	88.1
538361140	BN855_5890	326	senb	NC_022241	2fq1_B	285	88.4
538363806	BN855_32850	333	senb	NC_022241	No hit	-	-
161505744	SARI_03920	339	ses	NC_010067	No hit	-	-
161616799	SPAB_04618	339	spq	NC_010102	No hit	-	-
538364331	BN855_38160	339	senb	NC_022241	No hit	-	-
528818715	SN31241_20010	361	senn	NC_021902	1vh1_D	480	94
378960112	STBHUCCB_20620	361	sex	NC_016832	1vh1_D	479	94
16759510	Conserved	372	sty	NC_003198	No hit	-	-
56414314	SPA2188	372	spt	NC_006511	No hit	-	-
378698495	SL1344_0528	372	sey	NC_016810	No hit	-	-
378956078	SPUL_2424	372	sel	NC_016831	No hit	-	-
378444035	None	372	sev	NC_016854	No hit	-	-
383495341	UMN798_0581	372	sef	NC_017046	No hit	-	-
537437644	SPUCDC_2410	372	seg	NC_022221	No hit	-	-
549723245	Conserved	372	senr	NC_022544	No hit	-	-
550899973	Conserved	372	send	NC_022569	No hit	-	-
525841289	CFSAN001921_21865	384	set	NC_021814	No hit	-	-
525860398	CFSAN002050_25550	384	seeb	NC_021818	No hit	-	-
526221794	SE451236_02340	384	seen	NC_021820	No hit	-	-
525949065	SEEB0189_01285	384	see	NC_021844	No hit	-	-
529222678	I137_18460	384	seep	NC_021984	No hit	-	-
549482315	IA1_18065	384	sene	NC_022525	No hit	-	-
161502511	SARI_00555	465	ses	NC_010067	3oxp_B	147	44.2
161612923	SPAB_00629	465	spq	NC_010102	3oxp_B	147	44.2
378984906	STMDT12_C23650	465	sem	NC_016860	3oxp_B	147	44.2
16767539	STM4289	498	stm	NC_003197	2akl_A	110	68.2
16762971	Conserved	498	sty	NC_003198	2akl_A	110	68.2
29144458	t4196	498	stt	NC_004631	2akl_A	110	68.2
56416088	SPA4107	498	spt	NC_006511	2akl_A	110	68.2
62182738	SC4168	498	sec	NC_006905	2akl_A	110	68.2
161505231	SARI_03369	498	ses	NC_010067	2akl_A	92	66.3
161617431	SPAB_05288	498	spq	NC_010102	2akl_A	110	68.2
194444767	SNSL254_A4635	498	seeh	NC_011080	2akl_A	110	68.2
194448085	SeHA_C4635	498	seh	NC_011083	2akl_A	110	68.2
194735822	SeSA_A4544	498	sew	NC_011094	2akl_A	110	68.2
197365014	SSPA3814	498	sek	NC_011147	2akl_A	110	68.2
197249113	SeAg_B4551	498	sea	NC_011149	2akl_A	110	68.2
198243014	SeD_A4684	498	sed	NC_011205	2akl_A	110	68.2
205355060	SG4134	498	sega	NC_011274	2akl_A	110	67.3
207859443	SEN4060	498	setc	NC_011294	2akl_A	110	67.3
224586054	SPC_4352	498	sei	NC_012125	2akl_A	110	68.2
378702132	SL1344_4226	498	sey	NC_016810	2akl_A	110	68.2
378957845	SPUL_4281	498	sel	NC_016831	2akl_A	110	67.3
378962381	STBHUCCB_44380	498	sex	NC_016832	2akl_A	110	68.2
378447608	None	498	sev	NC_016854	2akl_A	110	68.2
378453234	STM14_5159	498	seo	NC_016856	2akl_A	110	68.2
378986964	STMDT12_C44240	498	sem	NC_016860	2akl_A	110	68.2
378991557	STMUK_4274	498	sej	NC_016863	2akl_A	110	68.2
383498867	UMN798_4648	498	sef	NC_017046	2akl_A	110	68.2
452121975	CFSAN001992_12425	498	senj	NC_020307	2akl_A	110	68.2
482906826	STU288_21535	498	setu	NC_021151	2akl_A	110	68.2
488656631	TY21A_21335	498	sent	NC_021176	2akl_A	110	68.2
525815577	SEEH1578_07620	498	seec	NC_021810	2akl_A	110	68.2
525828145	CFSAN002069_10645	498	senh	NC_021812	2akl_A	110	68.2
525839753	CFSAN001921_18970	498	set	NC_021814	2akl_A	110	68.2
525856209	CFSAN002050_04690	498	seeb	NC_021818	2akl_A	110	68.2
526218734	SE451236_04480	498	seen	NC_021820	2akl_A	110	68.2
525948743	SEEB0189_20995	498	see	NC_021844	2akl_A	110	68.2
529221780	I137_20500	498	seep	NC_021984	2akl_A	110	67.3
537439413	SPUCDC_4267	498	seg	NC_022221	2akl_A	110	67.3
549481441	IA1_20890	498	sene	NC_022525	2akl_A	110	68.2
549726803	Conserved	498	senr	NC_022544	2akl_A	110	68.2
550903633	Conserved	498	send	NC_022569	2akl_A	110	68.2

Later on, we performed a BLASTp search using 114 hypothetical sequences as ‘query’ and sequences of PDB as ‘database’. It was performed so that if there is any homology in already well characterized PDB database then it may lead us to classify the hypothetical proteins. The BLASTp showed hits against 81 queries with the PDB database while rest (i.e. 33) queries showed no hits (Table 5). The names of obtained hits for 81 queries were manually matched with the corresponding 24 COPs. The leftover 33 queries for which no similarity was found in PDB database were subjected to the bioinformatics tools i.e SVM–Prot and InterProScan. The obtained results for the 33 ‘no hits’ were confirmed by matching their names with the respective COPs. All results verified the output of CD-HIT clustering.

### 4. Validation from the literature

A similar study was performed by Becker et. al. using experimental techniques, so we have compared our results obtained from *in silico* approach. We also looked in the DrugBank of the possible entry of any drug target(s) against Salmonella. The DrugBank [35] reported 19 drug targets of *S*. *enterica*. 11 out of 19 belonged to the human, while remaining 8 belonged to the bacteria. The oxygen-insensitive NADPH Nitro reductase was common in 35 strains only. Other five did not belong to UMP. Only one (i.e. Penicillin-binding protein) out of 8 genes was present in the output of current strategy. Results are summarized in Table 6. Becker and his coworkers [20] have reported 155 essential enzymes for *S*. *enterica* serovar Typhimurium strain LT2, and compared those with various strains of *S*. *enterica* by performing extensive experimental study. We compared our identified 73 enzymes with the results of Becker and observed that 24 enzymes were shared by the reports of Becker et. al. (Table 3). Furthermore, the enzyme CheA (Chemotaxis Protein, COP # 49) was found as essential in current study while Backer et. al. suggested it as non-essential. This discrepancy may arise due to the recent updates in the DEG.

**Table 6:** *S*. *enterica* eight genes as drug targets–data from DrugBank.

Genes	Molecule	Output	Reason
16S rRNA	Nucleic Acid	excluded	Not the aim
30S ribosomal protein S10	Protein	X	Not in UMPs of SE
30S ribosomal protein S12	Protein	X	Not in UMPs of SE
DNA gyrase subunit A	Enzyme	X	Not in UMPs of SE
DNA topoisomerase 4 subunit A	Enzyme	X	Not in UMPs of SE
Oxygen-insensitive NADPH nitroreductase	Enzyme	X	In 35/42 strains
Penicillin-binding protein 2	Enzyme	present	Included
Probable pyruvate-flavodoxin oxidoreductase	Enzyme	X	Not in UMPs of SE

## Conclusion

We have performed extensive computational analysis of *S*. *enterica* at the level of core proteome to identify new potential drug targets. Subtractive proteomics through a novel approach was applied, i.e. by considering only proteins of the unique metabolic pathways of the pathogens and mining the proteomic data of all completely sequenced strains of the pathogen, thus improving the quality and application of the results. We identified 73 enzymes that are common to 42 strains of *S*. *enterica*, belong to unique metabolic pathways, are essential for pathogen survival and which have no human homologs. These four characteristics suggest that the enzymes are potential drug targets and should be tested experimentally. We compared them to experimental data [Becker et. al] showing that 24 out of the 73 (~33%) enzymes are current drug targets. The remaining 49 enzymes are new potential drug targets. We have annotated the function of 114 hypothetical proteins unique to S. enterica, providing additional new potential drug targets. Finally, our organization of the available core proteomic data (available in S2, S4, S5 and S6 Tables) in different categories e.g. clusters, organism codes, NCBI RefSeq IDs etc, provide a basis for further studies.

## Supporting Information

S1 Fig Strategy for subtractive proteomic analysis (XLSX)

S1 Table Details of downloaded biological datasets (XLSX)

S2 Table Number of Genes present in Unique Metabolic Pathways of 42 strains of S. enterica (XLSX)

S3 Table Discontinued and Updated NCBI GIs of Heidelberg CFSAN002069 and Typhi CT18 (XLSX)

S4 Table Cluster of Proteins (COPs) formed using CD-HIT (XLSX)

S5 Table Non-homologous Essential Enzymes of S. enterica 42 strains as drug targets (XLSX)

S6 Table Protein Identifiers and Names of 73 COPs (XLSX)

S1 Text Accessibility of NCBI GIs mentioned in S5 Table (DOCX)

## Abbreviations

KEGG: Kyoto Encyclopedia of Genes and Genomes
CD-HIT: Cluster Database at High Identity with Tolerance
DEG: Database of Essential Genes
UMP: Unique Metabolic Pathways
SVM: Support Vector Machine
KO: KEGG Orthology
FTP: File Transfer Protocol
NCBI-GI: National Center for Biotechnology Information—GenInfo Identifier
COP: Cluster of Proteins
API: Program Interface
BLAST: Basic Local Alignment Search Tool
BLASTp: Protein-Protein BLAST
VFDB: Virulence Factors Database
PDB: Protein Databank
SE: *Salmonella** enterica*
