# Supplementary material for: Core Proteomic Analysis of Unique Metabolic Pathways of Salmonella enterica for the Identification of Potential Drug Targets
Source: PLoS One. 2016 Jan 22;11(1):e0146796. doi: 10.1371/journal.pone.0146796 (PMC4723313; doi:10.1371/journal.pone.0146796)
Supplement: S1 Text — (DOCX) [file pone.0146796.s008.docx]

**S1 Text. Accessibility of NCBI GIs mentioned in S5 Table**

In our computational analyses, we have identified 73 enzymes belonging to 42 strains of *Salmonella enterica* as putative drug targets. Throughout our data mining, we had used NCBI-GI (an 8 or 9 digit number) as the identifier of each protein. These NCBI-GIs were taken from the complete proteome of *Homo sapiens* and respective *Salmonella enterica* strains downloaded from NCBI FTP server on **December 21, 2013**. After complete analyses using stand-alone bioinformatics tools and in-house Shell scripts, we tried to verify our results from online NCBI server, but **2,292** out of **3,105** NCBI-GIs (mentioned in S6 Table) were not accessible directly. Rather, a link to new Accession ID is mentioned. This occurred due to NCBI updated its FTP site in August 2014 under the project titled “Prokaryotic RefSeq Genome Re-annotation Project”,

<http://www.ncbi.nlm.nih.gov/refseq/about/prokaryotes/reannotation/>

<http://www.ncbi.nlm.nih.gov/news/08-26-2014-new-genomes-FTP-live/>

They have also modified FASTA format with NCBI-GI removed from the header.

<http://www.ncbi.nlm.nih.gov/news/09-17-2014-simple-FASTA-headers-genomes-FTP/>

For instance, if we search NCBI-GI “378699640” in NCBI Protein Database, following output appears;

The output contains link to

1. obsolete version “**Old YP_005181597.1**”
2. updated version “**New WP_000032881.1**”
3. BLAST result in **Identical Protein** Report
4. A comprehensive summary of **Re-annotation project**

So, one can have access to both obsolete and updated versions of the protein.
